# P-2035. Pharmacokinetics (PK) and Serum Virus Neutralizing Antibody (sVNA) Titers Following the 2^nd^ dose of Pemivibart in the Phase 3 CANOPY Trial

**DOI:** 10.1093/ofid/ofae631.2191

**Published:** 2025-01-29

**Authors:** Anna Holmes, Yong Li, Deepali Gupta, Chloe Katz, Mike Gavazzi, Pamela Hawn, Kathryn Mahoney, Myra Popejoy

**Affiliations:** Invivyd, Inc., Waltham, MA; Invivyd, Inc., Waltham, MA; Invivyd, Inc., Waltham, MA; Invivyd, Inc., Waltham, MA; Invivyd, Inc., Waltham, MA; Invivyd, Inc., Waltham, MA; Invivyd, Inc., Waltham, MA; Invivyd, Inc., Waltham, MA

## Abstract

**Background:**

Pemivibart (PEM) is a half-life extended recombinant human monoclonal IgG1 antibody that targets the SARS-CoV-2 spike protein receptor binding domain. PEM has been granted emergency use authorization (EUA) for the pre-exposure prophylaxis of COVID-19 in certain adults and adolescents with moderate-to-severe immune compromise, with dosing recommended every 3 months.

**Figure:**
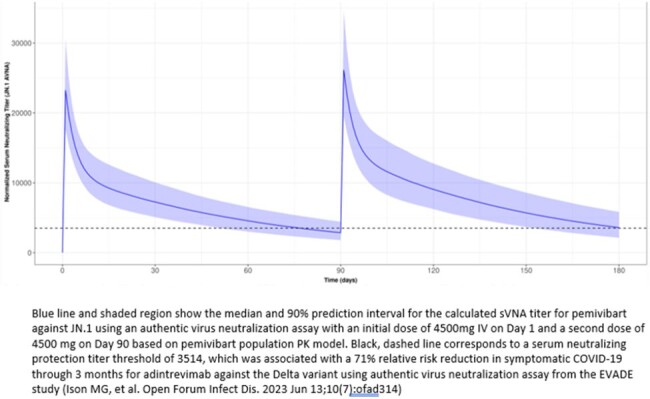

**Methods:**

CANOPY (NCT06039449) is a Phase 3 study investigating PEM 4500 mg for the prevention of COVID-19 that enrolled adult (≥18 years) participants (ppts) with immune compromise (Cohort A, single arm, open label) and those at risk of exposure to SARS-CoV-2 (Cohort B, randomized 2:1 to PEM or placebo). Ppts received an initial dose of study drug administered via intravenous (IV) infusion on Day 1 followed by a second dose approximately 3 months later (i.e., month 3). Serum samples were collected for PK analysis at the month 3 visit prior to and after receipt of the second PEM dose and at month 6 and month 12. Here we describe calculated sVNA titers for ppts in Cohort A following a second dose of PEM and further estimates from a population PK (PPK) model constructed with available data from a Phase 1 first-in-human study and the Phase 3 CANOPY study.

**Results:**

At the completion of the second (i.e., month 3) dose of PEM, the calculated sVNA geometric mean titer (GMT) to the SARS-CoV-2 Omicron JN.1 variant in Cohort A ppts was 17% higher than the corresponding value following the initial PEM dose. This is consistent with the estimated accumulation ratio based on the PPK model. Based on this model, the median calculated GMT is predicted to remain above the predefined protection titer threshold of 3514 for at least 90 days following dosing at month 3 (Figure 1). The steady-state AUC over the entire 3-month dosing interval is estimated to be approximately 30% higher than the AUC observed following the initial dose.

**Conclusion:**

A second dose of PEM 4500 mg IV given at month 3 boosted calculated sVNA titers and is anticipated to provide continued protection above predefined protection titer thresholds in immune compromised participants through 90 days post-dose for JN.1. The overall risk-benefit of PEM supports use in certain adults and adolescents with moderate-to-severe immune compromise for the prevention of COVID-19 per the EUA.

**Disclosures:**

Anna Holmes, PhD, Invivyd, Inc.: employee|Invivyd, Inc.: Stocks/Bonds (Private Company) Yong Li, PhD, Invivyd, Inc.: Employee|Invivyd, Inc.: Stocks/Bonds (Public Company) Deepali Gupta, BSc, Invivyd, Inc.: Employee|Invivyd, Inc.: Stocks/Bonds (Public Company) Chloe Katz, PMP, Invivyd, Inc.: Employee|Invivyd, Inc.: Stocks/Bonds (Public Company) Mike Gavazzi, B.S., Invivyd, Inc.: Employee|Invivyd, Inc.: Stocks/Bonds (Public Company) Pamela Hawn, Pharm.D., Invivyd, Inc.: Employee|Invivyd, Inc.: Stocks/Bonds (Public Company) Kathryn Mahoney, PharmD, Invivyd, Inc.: Employee|Invivyd, Inc.: Stocks/Bonds (Public Company) Myra Popejoy, Pharm.D., Invivyd, Inc.: employee|Invivyd, Inc.: Stocks/Bonds (Public Company)

